# Neuroimaging characteristics of myalgic encephalomyelitis/chronic fatigue syndrome (ME/CFS): a systematic review

**DOI:** 10.1186/s12967-020-02506-6

**Published:** 2020-09-01

**Authors:** Zack Y. Shan, Leighton R. Barnden, Richard A. Kwiatek, Sandeep Bhuta, Daniel F. Hermens, Jim Lagopoulos

**Affiliations:** 1 Sunshine Coast Mind and Neuroscience Thompson Institute, University of the Sunshine Coast, Birtinya, QLD 4575 Australia; 2 National Centre for Neuroimmunology and Emerging Diseases, Menzies Health Institute Queensland, Griffith University, Southport, QLD 4222 Australia; 3 Medical Imaging Department, Gold Coast University Hospital, Parklands, QLD 4215 Australia

**Keywords:** Chronic fatigue syndrome, Neuroimaging, Systematic review, Neurovascular coupling

## Abstract

**Background:**

Since the 1990s, neuroimaging has been utilised to study Myalgic Encephalomyelitis/Chronic Fatigue Syndrome (ME/CFS), a debilitating illness with unknown aetiology. While brain abnormalities in ME/CFS have been identified, relatively little is known regarding which specific abnormalities are consistently observed across research groups and to what extent the observed abnormalities are reproducible.

**Method:**

To identify consistent and inconsistent neuroimaging observations in ME/CFS, this retrospective and systematic review searched for studies in which neuroimaging was used to investigate brain abnormalities in ME/CFS in Ovid MEDLINE, PubMed (NCBI), and Scopus from January 1988 to July 2018. A qualitative synthesis of observations was performed to identify brain abnormalities that were consistently and inconsistently reported.

**Results:**

63 full-text articles were included in the synthesis of results from 291 identified papers. Additional brain area recruitment for cognitive tasks and abnormalities in the brain stem are frequent observations in 11 and 9 studies using different modalities from different research teams respectively. Also, sluggish blood oxygenation level-dependent (BOLD) signal responses to tasks, reduced serotonin transporters, and regional hypometabolism are consistent observations by more than two research teams. Single observations include abnormal brain tissue properties, regional metabolic abnormalities, and association of brain measures with ME/CFS symptoms. Reduced resting cerebral blood flow and volumetric brain changes are inconsistent observations across different studies.

**Conclusion:**

Neuroimaging studies of ME/CFS have frequently observed additional brain area recruitment during cognitive tasks and abnormalities in the brain stem. The frequent observation of additional brain area recruitment and consistent observation of sluggish fMRI signal response suggest abnormal neurovascular coupling in ME/CFS.

## Background

Myalgic Encephalomyelitis/Chronic Fatigue Syndrome (ME/CFS) is a complex and debilitating chronic illness. Patients with ME/CFS experience overwhelming fatigue that severely impacts their quality of life, with 25% of ME/CFS patients chronically bedridden or house-bound. However, there is no known underlying disease process, no biologically based treatment, and no objective diagnostic criteria for ME/CFS.

Although the aetiology of ME/CFS remains unclear, the well-documented neurological symptoms, sleep and autonomic dysfunction, abnormalities in cognition, and altered sensory and pain perception, suggest that abnormal brain function assumes a crucial role in the underlying disease process [1]. Given the aforementioned constellation of symptoms, ME/CFS has been classified as a neurological disease (ICD-10 G93.3; ICD-11 8E49) by the World Health Organization.

Researchers have used multiple neuroimaging techniques to explore structural, neurochemical, and functional brain changes in patients with ME/CFS since the 1990s. However, the results have been mixed for various reasons, such as small sample sizes and ill-defined disease classification. In short, it is recognised that abnormal brain function plays a critical role in ME/CFS. However, little is known about what abnormalities are consistently observed across multicentre studies and to what extent the observed abnormalities are reproducible. Therefore, there is a pressing need to summarise the consistency of observed abnormalities across studies in this field so as to inform future directions of neuroimaging research in ME/CFS.

This study systematically reviewed the neuroimaging studies of ME/CFS from January 1988 to July 2018 to specifically address the following three questions. (i) What structural or functional differences were consistently (i.e. by two or more research teams) observed in radiological brain imaging studies of ME/CFS? (ii) What brain measures were consistently associated with ME/CFS symptom phenotypes? (iii) What differences or symptoms and associated brain measures were inconsistently reported? The aim of this study was to facilitate a better-informed hypothesis of ME/CFS aetiology based on consistent findings, to reconcile some inconsistent findings, and to identify a future research focus.

## Methods

The objectives and analyses methods of this retrospective systematic review were specified in advance, documented and registered in the PROSPERO database [2]. The registration was submitted after the initial search but before the start of data extraction (examining contents of the selected papers).

### Data Source and Searches

An investigator (ZS) searched electronic databases, including Ovid MEDLINE, PubMed (NCBI), and Scopus, to identify relevant articles published between January 1988 (year of the first ME/CFS case definition) and July 2018. Cross-referencing supplemented the search during reviewing of full-text articles.

The search field included the title, abstract, and keywords with any combination of a population condition and an exposure condition as the search condition. The population condition included chronic fatigue syndrome, myalgic encephalomyelitis, systemic exertion intolerance disease, and their abbreviations. The exposure condition included neuroimage, neuroimaging, magnetic resonance imaging (MRI), functional magnetic resonance imaging, diffusion tensor imaging, magnetic resonance spectroscopy, arterial spin labelling, diffusion-weighted imaging, computed tomography, position emission tomography (PET), single-photon emission computed tomography (SPECT), ultrasound, and their abbreviations.

### Study Selection

We included the following studies: (i) peer-reviewed English language scientific journal articles; (ii) ME/CFS definitions including systemic exertion intolerance disease proposed by Institute of Medicine [3], International Consensus Criteria [4], Paediatric ME/CFS definition [5], Canadian consensus criteria [6], Centers for Disease Control and Prevention (CDC) Holmes criteria [7], London criteria [8], Reeves criteria [9], CDC Fukuda criteria [10], Oxford criteria [11]; iii) neuroimaging techniques of MRI, PET, SPECT, computed tomography (CT), ultrasound.

We excluded the following studies: (i) non-peer reviewed articles; (ii) articles not written in English; (iii) review, hypothesis without experimental data, editorial, and opinion articles, case reports and protocols, since this study is to summarise consistent observations; (iv) the modalities of electroencephalography, magnetoencephalography, and functional near-infrared spectroscopy because these techniques do not produce images and the scope of this study was limited to radiological brain imaging modalities.

Two investigators (ZS and LB) independently reviewed the abstract of each study to determine eligibility. Any disagreement was resolved by discussion to consensus.

### Risk of bias evaluation

The risk of bias for each study was evaluated using a tool adapted from QUADAS-2 [12], a quality assessment tool of diagnostic accuracy studies. This study reviewed observational studies; thus, the QUADAS-2 tool was adapted in 4 domains: (i) patient selection, (ii) index test, (iii) control standard, and (iv) control of confounding factors.

The signalling questions for the domain of patient selection included: (i) whether a consecutive or random sample of patients was enrolled; and (ii) whether the study avoided inappropriate exclusion.

The signalling questions for the domain of index test included: (i) whether a full brain imaging protocol was reported; and whether any potential bias existed in the protocol; ii) whether a significance threshold was pre-specified and whether the multiple comparison correction was appropriately handled.

The signalling questions for the domain of control standard included: (i) whether controls have similar age as patients or were controlled for age; (ii) whether the control group had the same gender ratio as the patient group or were controlled for gender ratio and (iii) whether controls were selected or recruited from the same or a similar population.

The signalling questions for the domain of control of confounding factors included: (i) whether psychiatric disorders including anxiety and depression were screened or controlled for; (ii) whether obesity was screened for, or body mass index was controlled for and (iii) whether neurologically active medication was screened.

Each domain of a study was summarised as low risk (no signalling question violated), moderate risk (one signalling question violated), or high risk (more than one signalling questions violated). If a study had one or more domain with high risks, the study results were interpreted with caution and relevant to the violations.

### Synthesis of results

The following contents were extracted from each article: author details, affiliations, country, ME/CFS diagnostic criteria, number of participants, demographics, aim of paper, image modality, analysis method, and observations.

Extracted findings were classified as frequent, consistent, single, and inconsistent observations. The frequent, consistent, and single observations were defined as same or similar findings reported by five or more research teams, by two to four teams, and one research team without inconsistent finding reported, respectively. The inconsistent observations were defined as findings reported by one or more research teams with contradictory findings reported. Studies that had one common author and from the same institution were considered as studies from the same research team.

## Results

**Fig. 1:**
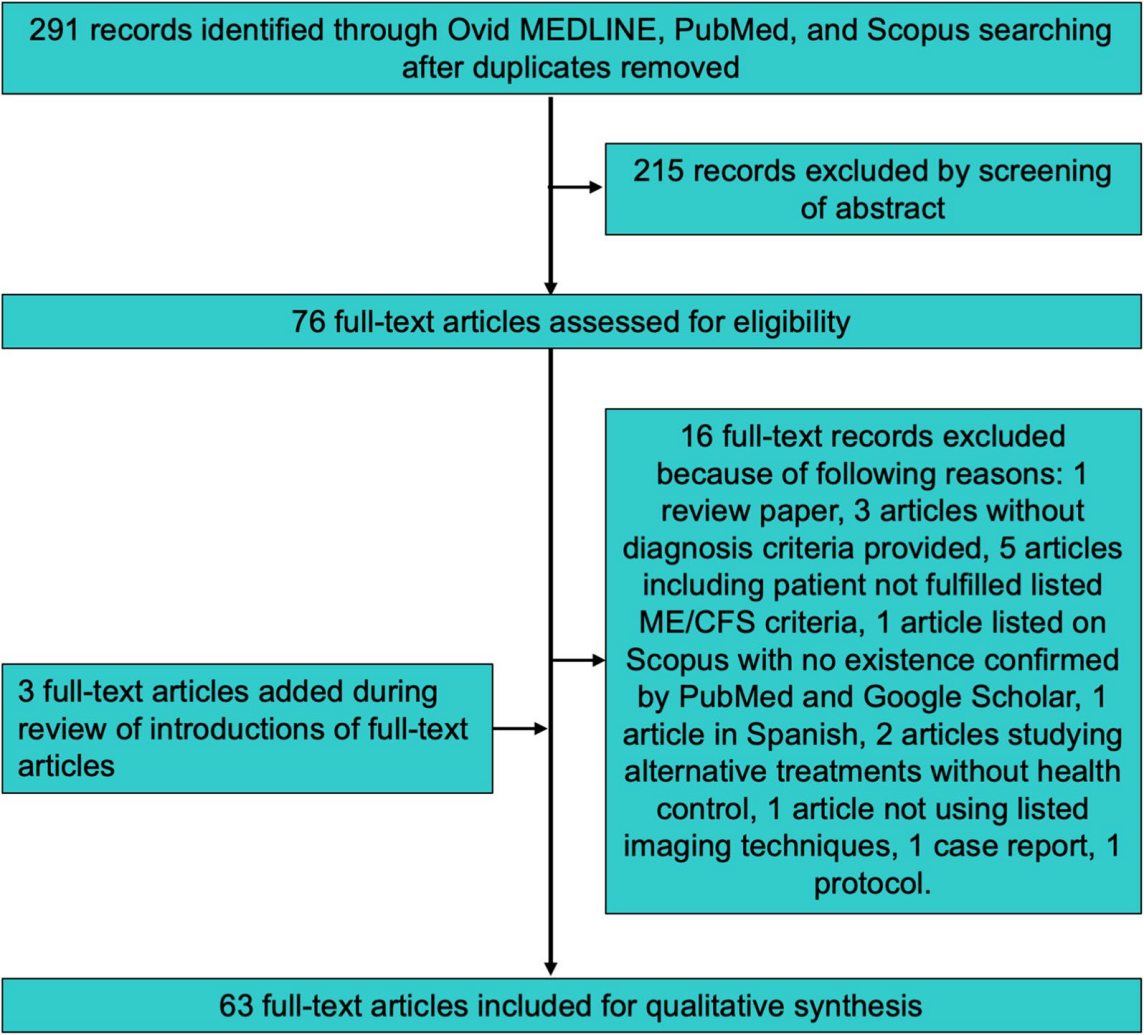
Summary of data source and study selection

Among 291 records identified by searches, 215 records did not meet the inclusion criteria and were excluded upon screening based on abstract and title. The full-text articles of the remaining 76 records were reviewed. Subsequently, 16 articles were excluded based on exclusion criteria. Three new articles were identified in the citations during the review of the full-text articles and were thus added (Fig. 1). Finally, 63 full-text articles were included in the synthesis of results.

### Study characteristics

**Fig. 2:**
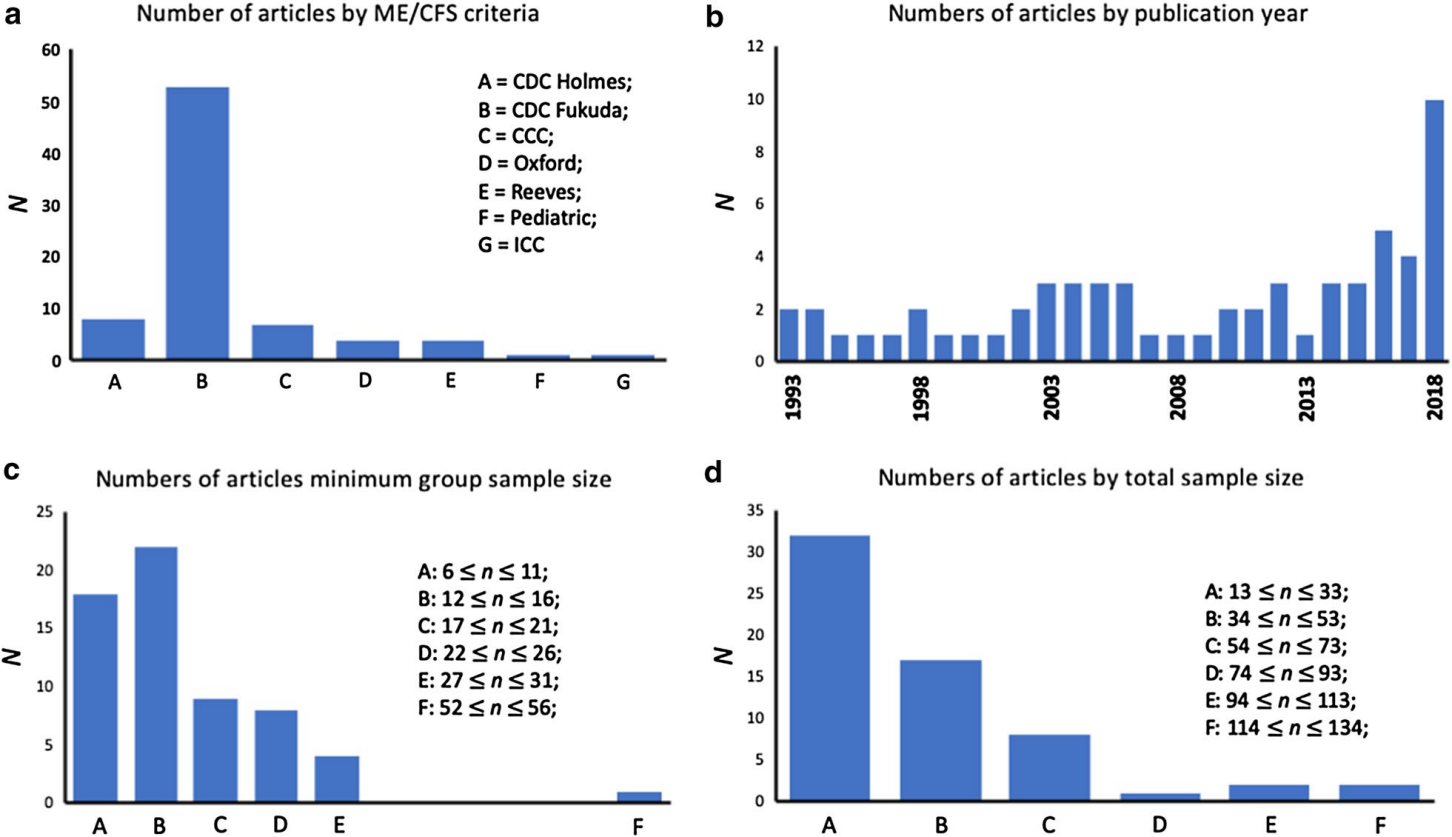
Absolute numbers of articles categorised by characteristics. **a** Numbers of articles using each myalgic encephalomyelitis/chronic fatigue syndrome. **b** Numbers of articles published each year. **c** Numbers of articles categorised by the sample size of either patient or control cohort, whichever is smaller. **d** Numbers of articles categorised by the sample size of the sum of patient and control cohort

The reviewed studies have used ME/CFS diagnostic criteria including CDC-Holmes criteria [7], Oxford criteria [11], CDC-Fukuda criteria [10], Canadian consensus criteria [6], Paediatric ME/CFS definition [5], Reeves criteria [9], and their combinations. Note that four studies [13–16] included in this review used a combination of CDC-Fukuda and Reeves criteria, another two studies [17, 18] used a combination of CDC-Holmes and Oxford criteria. The CDC-Fukuda definition is the most frequently used (54 out of 63 articles) criteria in the articles reviewed (Fig. 2a).

Although the number of articles published in recent years has appreciably increased (from approximate two articles per year before 2015 to six articles per year since), the overall number of radiological brain imaging studies of ME/CFS remains small with a maximum of 10 articles published in 2018 (Fig. 2b).

The sample size of the smaller group (patients or controls) in 40 out of the 63 articles reviewed were equal to or smaller than 16 (Fig. 2c). The total sample sizes (patients and controls) in 49 out of 63 articles reviewed were equal to or smaller than 53 (Fig. 2d).

The characteristics of ME/CFS diagnostic criteria, publication date, sample size, brain imaging modalities of each article are summarized in Additional file 1.

### Risk of bias

**Fig. 3:**
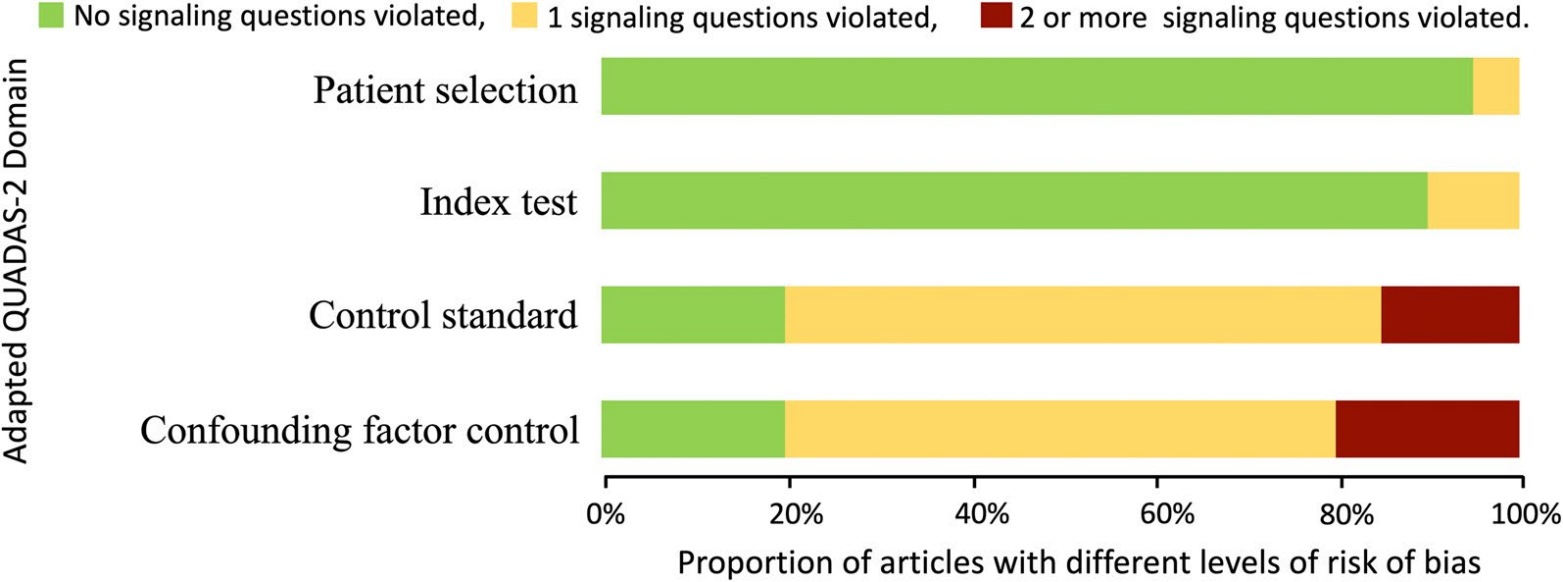
Summary of the risk of bias for studies reviewed. The risk of bias for each study was evaluated using a tool adapted from QUADAS-2, a quality assessment of diagnostic accuracy studies, for observational studies and listed in Additional file 1. The complete list of questions in each domain was detailed in the method section

With the adapted QUADAS-2, 61 and 56 out of 63 reviewed articles complied with all signalling questions in the domain of patient selection and index test, respectively. In the domain of control standard, 37 articles violated one signalling question, and 11 articles violated two more signalling questions. In the domain of confounding factor control, 35 articles violated one signalling question, and 14 articles violated two or more signalling questions (Fig. 3). The risks of bias of each article are summarized in SI.

### Frequent observations

#### Additional brain regions recruited for cognitive tasks in patients with ME/CFS

A SPECT study reported that patients with ME/CFS presented more diffuse and less focal activation patterns than controls when performing the paced auditory serial addition test [19]. We identified 12 task functional MRI studies of ME/CFS [14, 16, 20–29]. Different patterns of blood oxygenation level-dependent (BOLD) signal changes associated with tasks were reported in each article (detailed in SI). Of these 12 studies, 10 articles reported that patients with ME/CFS either recruited additional brain regions or had a greater BOLD response than controls for cognitive tasks but with the same performance [16, 20–25, 27–29], although decreased BOLD activity was also reported in specific regions. Two remaining studies tested basal ganglia function [14] and reward sensitivity [26] and observed lower BOLD changes in basal ganglia in gambling tasks [14] and lower BOLD changes in the putamen in low-reward condition gambling task [26].

#### Abnormalities identified in the brain stem of patients with ME/CFS

Five studies from two groups reported structural abnormalities in the brain stem in ME/CFS using MRI [30–34]. Additionally, higher binding potential values of 11C-(R)-(2-chlorophenyl)-N-methyl-N-(1-methylpropyl)-3-isoquinoline-carboxamide, indicating local neuroinflammation, was observed in the brain stem in ME/CFS using PET [35]. Three studies reported hypoperfusion in the brain stem using^99^Tc^m^-hexamethyl-propylene-aminoxime (^99^Tc^m^-HMPAO) SPECT [17], [2-^11^C] acetyl-L-carnitine PET [36], and 18-fluorodeoxyglucose (FDG) PET [37]. However, two ^99^Tc^m^-HMPAO SPECT studies from another research group did not observe significant regional cerebral blood flow (CBF) differences in the brain stem in ME/CFS [38, 39].

### Consistent observations

#### Sluggish BOLD responses to tasks

Shan et al. examined sample entropy, a measure of the complexity of physiological time-series signals, of BOLD signal changes associated with the Stroop task in ME/CFS [29]. BOLD sample entropies in 10 regions were significantly lower in ME/CFS and explained 56% of variances in the 36-item Short Form Health Survey scores [29]. Another fMRI study observed that responsiveness of the auditory cortex was attenuated in patients with ME/CFS. In addition, the rate of the attenuation was positively correlated with the subjective sensation of fatigue [40].

#### Reduction of serotonin transporters in ME/CFS

A widespread reduction in the serotonin 1A receptor binding potential, particularly in the hippocampus bilaterally [41], and reduced serotonin transporters density in the rostral subdivision of the anterior cingulate [42] were reported from two separate groups.

#### Regional reduced FDG uptakes

FDG PET from two different groups showed significant hypometabolism in the right mediofrontal cortex and brain stem [37] and in the orbitofrontal cortex [43].

### Single observations

#### Regional brain tissue characteristics

Using radiologists’ reporting, abnormal MRI scans were found to be correlated with low physical functions [44]; more abnormal scans were found in patients with ME/CFS but no psychiatric diagnosis, than in those with psychopathy and controls [45]; and patients with ME/CFS had more abnormal scans than healthy controls (HCs) [46]. Significantly different T1 weighted and T2 weighted spin echo signal intensities were found in various brain regions using voxel based comparison and regression analysis (detailed in SI) [30, 47, 48]. However, these abnormalities were distributed in different regions in different studies.

#### White Matter microstructure by diffusion tensor imaging

Zeineh et al. observed a significant increase of fractional anisotropy accompanied by an increase in axial diffusivity and a decrease in radial diffusivity in the right anterior arcuate fasciculus in patients with ME/CFS [49].

#### Differences in brain metabolites

Using MR spectroscopy, lower N-acetylaspartate concentration in the hippocampus [50], lower cortical glutathione levels [51, 52], higher choline concentration in the basal ganglia [53], higher lactate concentrations in the lateral ventricles (from a same group) [51, 52, 54–56], higher choline to creatine ratio in the occipital cortex [57] have been reported in ME/CFS. No differences in $\gamma$-aminobutyric acid nor glutamate + glutamine either in the occipital cortex or anterior cingulate cortex [55] or N-acetylaspartate to creatine ratio in the frontal and occipital cortex [57] have been reported. More pain was associated with a reduced N-acetylaspartate to creatine ratio, however, no group difference was found in the dorsolateral prefrontal cortex [15]. Yamamoto et al. reported that patients with ME/CFS and positive serum autoantibodies showed significantly lower brain muscarinic cholinergic receptor binding, although the acetylcholinesterase activity remained similar [58].

#### Intracranial compliance measured as a ratio of changes in intracranial volume and pressure during the cardiac circle

There was no group difference, although low intracranial compliance and high cerebral perfusion were associated with increased severity of symptoms of orthostatic intolerance in patients with ME/CFS [59].

#### Association of cerebral vascular control with skeletal muscle pH in ME/CFS

He et al. reported that cerebral vascular control measured by Valsalva manoeuvre fMRI is closely related to skeletal muscle pH both at rest and after dynamic stimulation in ME/CFS [60]. However, this study did not include a HC group comparison [60].

### Inconsistent observations

#### Global grey matter (GM) and white matter (WM) volumes

Five studies have reported no significant difference in global GM or WM volumes [31, 33, 47–49]. A one-year longitudinal study reported that the mean and the longitudinal change of cerebrospinal fluid (CSF) volume was not significantly different between ME/CFS patients and HCs [61]. De Lange et al. observed a reduction in total GM volumes but no difference in total WM volumes in ME/CFS [62]. Finkelmeyer et al. reported higher total GM and smaller WM volumes after accounting for total intracranial volume in ME/CFS [34].

#### Regional GM and WM volumes

De Lange et al. reported that there was no significant difference in regional GM or WM volumes in ME/CFS [62]. Finkelmeyer et al. observed increased regional GM volumes in the amygdala and insula and decreased regional WM volume in the midbrain, pons, and right temporal lobe [34]. Two studies from different groups reported reduced regional GM volumes in various brain regions [63, 64]. A 6-years longitudinal study observed a significant decrease of WM volume in the left inferior fronto-occipital fasciculus (IFOF) in ME/CFS while it was unchanged in HCs [47]. Significantly lower WM volume in the left IFOF/arcuate was observed in a different sample by the same group [48]. Zeineh et al. observed significantly lower supratentorial WM volumes, statistically equivalent regional GM volumes and cortical thickness in the left hemisphere, and higher cortical thickness in the right lateral occipital, precentral, middle temporal, postcentral and pars orbitalis in ME/CFS [49]. Sevel et al. used a support vector machine based on 61 anatomical features (indices of area, thickness, and volume of cortical and subcortical structures) to classify ME/CFS with 79.58% accuracy [65].

#### Resting cerebral blood flow (CBF)

Eight studies observed decreased global or regional CBF in ME/CFS [17, 18, 51, 52, 66–69]. Peterson et al. observed reduced cerebral perfusion in 3 of 10 at rest and 6 of 10 patients with ME/CFS after an exercise of walking 1.61 km/hour for 30 min while only in 2 of 10 HCs at rest and post-exercise [70]. However, another six studies observed no global or regional CBF difference [38, 59, 61, 71–73]. Fischler et al. observed three regions of interest (ROIs) with hypoperfusion and 9 ROIs with hyper perfusion out of 45 ROIs [39] in ME/CFS. Gay et al. observed no global CBF difference but reduced regional resting CBF in occipital and temporal lobes [74].

#### Functional connectivity (FC)

Abnormal FC in the affective network, salience network, default mode network (DMN) were reported using ASL and fMRI [13, 28, 74–76]. Two resting state studies found increased FC in the DMN between posterior cingulate cortex (PCC) and ACC [13, 75] while two studies did not observe a FC difference between PCC and ACC [28, 74] in ME/CFS. Gay et al. observed reduced FC between PCC and ACC during cognitive tasks [74]. Two studies from the same research team observed reduced FC from the PCC to the salience network [74, 75].

## Discussion

The synthesis of the reviewed studies generated two frequent observations (larger recruitment of brain regions during cognitive tasks and abnormalities in the brain stem), three consistent observations (sluggish fMRI signal response to tasks, reduced serotonin transporters, and regional hypometabolism), and five single observations (regional T1 and T2 spin-echo intensity differences and abnormal clinical correlations, white matter microstructural changes, regional metabolite abnormalities, associations between intracranial compliance and severity of orthostatic intolerance symptoms, and associations between cerebral vascular control and skeletal muscle pH).

Larger recruitments of brain areas could be potentially explained by the sluggish BOLD response to tasks, which was observed consistently in two fMRI studies [29, 40]. The BOLD response is determined by neurovascular coupling (NVC) which consists of an initial feedforward mechanism of glutamate activation of a Ca^2+^ dependent signalling pathway in both neurons and astrocytes to release vasoactive factors to increase local blood flow, and secondary feedback driven by metabolism [77]. Several recent studies have demonstrated that Ca^2+^ mobilisation is impaired in ME/CFS from genetic [78, 79], molecular biological [80], and electrophysiological aspects [81]. These observations would suggest, it is plausible that glutamate-Ca^2+^ NVC pathways in patients with ME/CFS may be impaired because of reduced TRPM3 activity and Ca^2+^ mobilisation.

Impaired NVC could have multiple detrimental effects on the brain. Firstly, deficits in matching local CBF to neuronal activity may lead to hypoxia. Secondly, inadequate energy supply to activated neurons may potentially lead to energy failure of ionic pumps. Thirdly, oxidative stress that impairs blood vessel endothelial cell function may cause blood–brain barrier (BBB) breakdown and neuroinflammation. These potential detrimental effects are generally diffuse and multifocal in the brain, leading to reduced cognitive efficiency in ME/CFS and manifesting as more extensive functional recruitment during cognitive task performance. A similar manifestation has been observed in patients with other neuropathologies that result in diffuse damage such as traumatic brain injury [82]. Reduced cognitive efficiency may be the neurological underpinning of the observation that patients with ME/CFS often report subjective changes when performing cognitive tasks despite normal objective performance. Impaired NVC in ME/CFS is further supported by structural MRI observations of regional white matter loss in the left inferior fronto-occipital fasciculus [47] and the brain stem [30–34], both being brain regions sensitive to hypoxia [83, 84].

Dysfunction of NVC is closely related to neuroinflammation, which is another neurological disease process proposed to underly ME/CFS [85, 86]. Dysfunction of NVC may trigger neuroinflammation. The typical NVC dysfunction features of inflammatory and angiogenic activation triggered by hypoxia may jointly result in a leaky BBB, brain oedema, and neuronal dysfunction and damage [87]. Vice versa, neuroinflammation and infection may result in NVC dysfunction, whereby, activation of microglia, especially the formation of the M2 phenotype, exacerbates damage to the BBB. Neuroinflammation molecular patterns can interact with neurotransmitters, increase procoagulant activity and thrombosis, and cause endothelial injury and damage, resulting in NVC dysfunction [88]. Although there is no direct evidence to support either dysfunction of NVC or neuroinflammation as the primary aetiology factor of ME/CFS, we posit that dysfunction of NVC constitutes a critical underlying disease process in ME/CFS.

The synthesis of articles in this review also generated three consistent observations: sluggish BOLD response to task [29, 40], reduced serotonin transporter, and regional hypometabolism. The sluggish BOLD response is consistent with NVC dysfunction. Two studies observed reduced serotonin transporter in ME/CFS [41, 42]. Serotonergic imbalance is a prominent feature in major depression [89] and a large percentage of patients with ME/CFS experience depression. These observations may help explain the well documented depressive symptoms in ME/CFS. The evidence of regional hypometabolism [37, 43] is aligned with an NVC dysfunction. The hypoxia, oxidative stress, and energy deficiency caused by NVC dysfunction may lead to reduced glucose uptake and result in a vicious cycle [90]. The inconsistent observations of volumetric differences in ME/CFS are consistent with the notion that the detrimental effects of NVC dysfunctions are diffuse and multifocal in the brain. Different studies may generate positive or negative findings depending on the disease severity and patient selection.

This review noted ME/CFS as an under-recognised disease and that brain imaging studies of ME/CFS suffered from small sample size. A small sample size not only reduces the chance of detecting a true effect but also reduces the likelihood that a statistically significant result reflects a true effect [91]. As such, we argue that there is a pressing need to establish a collaborative neuroimaging databank for ME/CFS in addition to a proposed ME/CFS biobank [92]. Notably, more than 80% of reviewed studies did not control for lifestyle differences. Physical activity may impact brain structures and functions [93, 94]. Given that patients with ME/CFS have to reduce their daily activities because of fatigue, objective measurement of physical activity in both patients and controls is essential to differentiate between brain differences that are unique in ME/CFS and those due to reduced physical activity.

This review was unable to perform a formal meta-analysis because: (i) There were few studies on neuroimaging of ME/CFS. (ii) Each study used an ad hoc analysis method. (iii) Different aspects were investigated in different studies. Nevertheless, this systematic review of neuroimaging in ME/CFS provides a snapshot of ME/CFS brain imaging studies to date and identifies deficiencies that need to be accounted for in future studies.

## Conclusion

We systematically reviewed 63 scientific articles on brain imaging studies of ME/CFS. The results of our qualitative synthesis of these articles are consistent with autonomic dysfunctions in ME/CFS, potentially arising centrally. In addition, this review highlighted that more extensive brain areas were recruited during cognitive tasks in patients with ME/CFS, which had not previously been well recognised. This feature may represent brain function inefficiency caused by an NVC dysfunction. This review also identified deficiencies in the previous neuroimaging studies in ME/CFS, including small sample sizes and lack of objective physical activity measurement in control groups. We advocate establishing a neuroimaging databank of ME/CFS to mitigate these issues.

## Supplementary information

Supplementary file1 (DOCX 401 kb)

## Data Availability

The articles reviewed in this study are available in the public domain.

## Abbreviations

ACC: Anterior cingulate cortex
BBB: Blood–brain barrier
BOLD: Blood oxygenation level-dependent
CBF: Cerebral blood flow
CDC: Centers for Disease Control and Prevention
DMN: Default mode network
FC: Functional connectivity
FDG: 8-fluorodeoxyglucose
GM: Grey matter
HC: Health control
ME/CFS: Myalgic encephalomyelitis/chronic fatigue syndrome
NVC: Neurovascular coupling
PCC: Posterior cingulate cortex
SI: Support Information
WM: White matter
