# Supplementary material for: Neuroimaging characteristics of myalgic encephalomyelitis/chronic fatigue syndrome (ME/CFS): a systematic review
Source: J Transl Med. 2020 Sep 1;18:335. doi: 10.1186/s12967-020-02506-6 (PMC7466519; doi:10.1186/s12967-020-02506-6)
Supplement: Supplementary file 1 — Supplementary file1 (DOCX 401 kb) [file 12967_2020_2506_MOESM1_ESM.docx]

**Additional Information**

1. Characteristics of reviewed individual neuroimaging studies in myalgic encephalomyelitis/chronic fatigue syndrome
2. Risk of bias of reviewed individual neuroimaging studies in myalgic encephalomyelitis/chronic fatigue syndrome (ME/CFS)
3. Observations of reviewed individual neuroimaging studies in myalgic encephalomyelitis/chronic fatigue syndrome (ME/CFS)

**Table S1 Characteristics of reviewed individual neuroimaging studies in myalgic encephalomyelitis/chronic fatigue syndrome (ME/CFS)^§^**

| Study | Institute | Country | Year | Diagnostic Criteria | *N* | Age | Aim | Modality | Analysis |
| --- | --- | --- | --- | --- | --- | --- | --- | --- | --- |
| Barnden et al [30] | Griffith University | Australia | 2014 | Fukuda 1994 and Canadian 2003 | 25; 25 | 32 (19 -46); 32.8 (20 - 46) | To investigate white matter (WM) involvement in ME/CFS | sMRI | SPM5 voxel wise regression |
| Barnden et al [31] | The Queen Elizabeth Hospital | Australia | 2011 | Fukuda 1994 and Canadian 2003 | 25; 25 | 32 (19 -46); 32.8 (20 - 46) | To explore brain involvement in chronic fatigue syndrome | sMRI | SPM5 voxel wise group comparison and regression |
| Barnden et al [32] | Griffith University | Australia | 2016 | Fukuda 1994 and Canadian 2003 | 25; 25 | 32 (19 -46); 32.8 (20 - 46) | To investigate autonomic function correlation with brain MRI measures | sMRI | SPM5 voxel wise regression and group comparison |
| Barnden et al [33] | Griffith University | Australia | 2018 | CDC-Fukuda 1994 | 43; 27 | NP | To investigate myelin and Fe concentration in ME/CFS | sMRI | SPM12 voxel wise regression and group comparison |
| Biswal et al [66] | UMDNJ-New Jersey Medical School | USA | 2011 | CDC-Fukuda 1994 | 11; 10 | NP | To determine if earlier finding of reduced cerebral blood flow (CBF) can be replicated using alternative method and to determine if there are individual differences in CBF among ME/CFS patients | ASL MRI | AFNI and in-house analysis tools |
| Boissoneault et al [75] | University of Florida | USA | 2016 | CDC-Fukuda 1994 | 17; 17 | 49.83 ± 11.32; 48.88 ± 12.00 | To examine the functional network changes of individuals with ME/CFS | ASL MRI | SPM12, ASLtbx, Conn seed to voxel connectivity analysis |
| Boissoneault et al [76] | University of Florida | USA | 2018 | CDC-Fukuda 1994 | 19;15 | 48.26 ± 12.22; 47.87 ± 12.14 | To examine both static and dynamic connectivity changes in FC among several a priori selected brain regions during a fatiguing cognitive task. | ASL MRI | SPM12, ASLtbx, Conn seed to voxel static connectivity analysis and dynamic connectivity |
| Boissoneault et al [71] | University of Florida | USA | 2018 | CDC-Fukuda 1994 | 14;14 | 48.57 ± 12.11; 49.57 ± 13.16 | To examine whether variability in cerebral blood flow (CBFV) and heart rate (HRV) are associated with fatigue level in ME/CFS | ASL MRI | ECG data processed using Impresario, MRI processed using SPM12, ASLtbx, and REX |
| Brooks et al [50] | University of Liverpool | UK | 2000 | CDC-Holmes 1988 | 7;10 | 26 - 40; 21-41 | To explore hippocampus volumes and metabolite concentrations in ME/CFS | sMRI & MRS | Analyse for morphometric measures and VARPRO in MRUI for MRS |
| Caseras et al [20] | King’s College London | UK | 2006 | CDC-Fukuda 1994 | 17; 12 | 33.53 ± 6.17; 33.50 ± 7.12 | To examine the neural correlates of working memory in patients with ME/CFS compared with controls. | block design fMRI | XBAM general linear modelling |
| Caseras et al [21] | King’s College London | UK | 2008 | CDC-Fukuda 1994 | 12; 11 | 33.75 ± 7.64; 34.36 ± 6.77 | To explore the functional brain response, using fMRI, to the imaginal experience of fatigue in ME/CFS patients and controls | block design fMRI | XBAM general linear modelling |
| Chaudhuri et al [53] | University of Glasgow | UK | 2003 | CDC-Fukuda 1994 | 8; 8 | 42 (32 - 54); 42.5 (28 - 59) | To study the metabolic functions of the basal ganglia in ME/CFS to test the hypothesis that fatigue in ME/CFS may have a neurogenic component. | Singe voxel 1H MRS | Peak area comparison after phase and baseline correction |
| Cleare et al [41] | Imperial College School of Medicine | UK | 2005 | Oxford 1991 & CDC-Fukuda 1994 | 10;10 | 46.5 ± 5.9; 40.7 ± 10 | To assess 5-HT function in ME/CFS | [11C] WAY-100635 PET | ROI based analysis |
| Cook et al [44] | University of Wisconsin – Madison | USA | 2017 | CDC-Fukuda 1994 & CCC 2003 | 15; 15 | 42.7 ± 11.1; 43.2 ± 10.4 | To determine the neural consequences of acute exercise using functional brain imaging | Two block design fMRI sessions before and after exercise | AFNI general linear model |
| Cook et al [22] | University of Wisconsin – Madison | USA | 2007 | CDC-Fukuda 1994 | 9; 11 | 43 ± 8; 42 ± 9 | To use fMRI to determine the association between feelings of mental fatigue and BOLD brain responses during a mentally fatiguing cognitive task. | block design fMRI | SPM2 general linear model |
| Costa et al [17] | UCL Medical School | UK | 1995 | CDC-Holmes 1988 & Oxford criteria | Initial study: 24, 24; Further study 43, 16 HC, 20 MDD | 36 ± 13, 31 ± 10; 19 - 76, 24 - 52, 18 - 52 | To investigate brain perfusion abnormalities in ME/CFS | ^99^Tc^m^-HMPAO brain SPECT | ROI based qualitative analysis and quantitative analysis |
| De Lange et al [62] | Radboud University Nijmegen | The Netherlands | 2005 | CDC-Fukuda 1994 | 13;15 & 15; 13 | 28.9 ± 6.1; 25.7 ± 6.5 & 43.9 ± 14.4; 43.4 ± 14.1 | To investigate brain morphology in ME/CFS patients using VBM | sMRI | SPM2 VBM |
| De Lange et al [24] | University Nijmegen | The Netherlands | 2004 | CDC-Fukuda 1994 | 16;16 | 28.4 ± 6; 24.9 ± 6.4 | To investigate the behavioural and neural correlates of movement planning in ME/CFS patients | event related fMRI | SPM99 general linear model |
| Finkelmeyer et al [59] | Newcastle University | UK | 2018 | CDC-Fukuda 1994 | 40;10 | 45.3 ± 11.6; 49.4 ± 15.3 | To determine intracranial compliance in ME/CFS | phase-contrast, quantitative flow MRI & FAIR ASL MRI | In-house toolkit for analysis of Intracranial compliance (ICC) analysis and SPM8 assisted ASL processing |
| Finkelmeyer et al [34] | Newcastle University | UK | 2018 | CDC-Fukuda 1994 | 42;28 | 45.6 ± 11.7; 48.4 ± 11.3 | To investigate global and regional GM and WM in ME/CFS using VBM | sMRI | CAT12 in SPM12, VBM |
| Fischler et al [38] | University Hospital KU Leuven | Belgium | 1996 | CDC-Holmes 1988 | ME/CFS, MDD, HC: 16, 19, 20 | 35 ± 9.6; 40 ± 12.1; 36 ± 8.6 | To examine rCBF and its relationship with symptoms in ME/CFS | ^99^Tc^m^-HMPAO brain SPECT | Semiquantitative ROI based analysis |
| Fischler et al [39] | University Hospital KU Leuven | Belgium | 1998 | Oxford 1991 & CDC-Fukuda 1994 | 22; 15 | 34.7 ± 8.3; 28.5 ± 5.2 | To confirm and investigate rCBF in ME/CFS | ^99^Tc^m^-HMPAO brain SPECT | Semiquantitative ROI based analysis |
| Gay et al [74] | University of Florida | USA | 2016 | CDC-Fukuda 1994 | 19;17 | 52.33 ± 10.63; 48.75 ± 11.75 | To investigate the association between fatigue and altered resting-state FC in myalgic encephalomyelitis/chronic fatigue syndrome (ME/CFS). | resting state ASL and BOLD fMRI | ASLtbx and SPM8, SPM12 for ASL; GIFT, SPM12, Conn for fMRI |
| Goldberg et al [67] | Harbor_UCLA Medical Center | USA | 1997 | CDC-Holmes 1988 & CDC-Fukuda 1994 | 13; 13 | 14.1 (9 - 18); 9.3 ± 3.2 | NeuroSPECT findings in 13 ME/CFS children | Xe 133 and ^99^Tc^m^-HMPAO brain SPECT | Quantitative comparison (linear regression to convert HMPAO to absolute rCBF using Xe data) |
| He et al [60] | Newcastle University | UK | 2013 | CDC-Fukuda 1994 | 17;0 | NP | To explore whether there was a physiological link between cerebral vascular control and skeletal muscle pH management in ME/CFS. | Resting ASL, block design the Valsalva manoeuvre fMRI, muscle MRS | SPM8 |
| Ichise et al [68] | University of Toronto | Canada | 1992 | CDC-Holmes 1988 | 60;14 | 32 ± 2; 36 ± 1 | To assess regional CBF in ME/CFS | ^99^Tc^m^-HMPAO brain SPECT | in-house toolkit |
| Kim et al [13] | Yonsei University | Korea | 2015 | CDC-Fukuda 1994 & Reeves 2005 criteria | 18; 18 | 45.9 ± 3.2; 43.9 ± 4.8 | To evaluate alterations of resting-state functional connectivity in ME/CFS patients. | Resting state fMRI | SPM8 Conn |
| Kuratsune et al [36] | Osaka City University Graduate School of Medicine | Japan | 2002 | CDC-Fukuda 1994 | 8;8 | 43.3 ± 7.4; 40.7 ± 6.7 | To study the cerebral uptake of acetyl carnitine in ME/CFS | [2-^11^C] acetyl-L-carnitine PET | autoradiographic method |
| Lange et al [45] | UMDNJ-New Jersey Medical School | USA | 1999 | In-house modified CDC-Fukuda 1994 | 39;19 | 38.1 ± 7.8; 36.9 ± 5.9 | To determine the profile of MRI abnormalities in ME/CFS | T1 and T2 spin echo MRI | Two radiologist evaluation |
| Lange et al [25] | UMDNJ-New Jersey Medical School | USA | 2005 | CDC-Fukuda 1994 | S1*: 6; 7  S2*: 19:15 | S1: 38.17 ± 9.0; 30.71 ± 9.6; S2: 37.53 ± 8.0; 30.87 ± 7.5 | To test to which extent, ME/CFS affects BOLD signal change during cognitive performance | block design fMRI | SPM99 general linear model |
| Lewis et al [72] | University of Washington | USA | 2001 | CDC-Fukuda 1994 | 22;22 | 41.4 (19 - 57) | To evaluate the relationship between regional cerebral blood flow (rCBF) and the disease in monozygotic twins discordant for ME/CFS. | ^99^Tc^m^-HMPAO brain SPECT | Examination from two experienced radiologists and semiquantitative analysis |
| Mathew et al [54] | Weill Medical College of Cornell University | USA | 2009 | CDC-Fukuda 1994 | ME/CFS; GAD; HC: 16; 14; 15 | 37.6 ± 9.9; 37.9 ± 14.2; 35.3 ± 10.3 | To compare lateral ventricular volume and lactate concentration among ME/CFS, GAD, and HC | T1 SPGR MRI and proton MRSI | Medx and Slicer for ventricular volume and in-house MRS analysis tool |
| Miller et al [14] | Emory University School of Medicine | USA | 2014 | CDC-Fukuda 1994 & Reeves 2005 criteria | 18;41 | 44.2 ± 11.1; 47.2 ± 9.2 | To test the hypothesis of decreased basal ganglia function in ME/CFS | fMRI | AFNI fMRI ROI (caudate, putamen and globus pallidus) analysis |
| Mizuno et al [26] | RIKEN Center for Life Science Technologies | Japan | 2016 | Jason 2006 pediatric ME/CFS | 13;13 | 13.6 ± 1.0; 13.7 ± 1.3 | to determine whether brain activity in regions related to reward sensitivity is impaired in CCFS patients. | block design fMRI | SPM8 two level general linear modelling with prior ROIs (caudate, putamen, and thalamus) |
| Mizuno et al [27] | RIKEN Center for Life Science Technologies | Japan | 2015 | CDC-Fukuda 1994 | 15;13 | 13.5 ± 1.0; 13.4 ± 1.2 | To identify the neural relationship between fatigue and divided attention in HCA and CCFS patients. | The Kana Pickout Test (KPT) block design fMRI | SPM5 two level general linear modelling |
| Murrough et al [55] | Weill Medical College of Cornell University | USA | 2010 | CDC-Fukuda 1994 | ME/CFS; MDD; HC: 17; 21; 19 | 47.9 ± 9.3; 39.1 ± 11.1; 37.2 ± 13.8 | To assess ventricular lactate levels in ME/CFS compared with health control and major depressive disorder (MDD) | MRSI for ventricular lactate and PRESS MRS for GABA and Glx | in-house analysis toolkit |
| Nakatomi et al [35] | RIKEN Center for Life Science Technologies | Japan | 2014 | CDC-Fukuda 1994 & ICC 2011 | 9;10 | 38.4 ± 5.1; 39.1 ± 6.0 | To investigate the existence of neuroinflammation in ME/CFS patients. | ^11^C- (R)-PK11195 PET | in-house linear graphic analysis |
| Natelson et al [46] | UMDNJ-New Jersey Medical School | USA | 1993 | CDC-Holmes 1988 | 52;52 | 37.7 (16 - 56); 37.4 (16 - 56) | To test if there are MR abnormalities in ME/CFS | 2D T1 and T2 images | Two radiologists blindly review and third one as "tie-breaker" in 5 cases |
| Natelson et al [56] | Mount Sinai Beth Israel | USA | 2017 | CDC-Fukuda 1994 (in-house adaptation) | ME/CFS only; ME/CFS/FM; FM only, HC: 17, 21, 7, 29 | NP | To investigate CSF lactate levels in ME/CFS, FM, ME/CFS and FM patients compared with HCs | ^1^H MRSI | in-house analysis toolkit |
| Natelson et al [52] | Mount Sinai Beth Israel | USA | 2017 | CDC-Fukuda 1994 (in-house adaptation) | ME/CFS-NP (without psychiatric diagnosis): 27, ME/CFS-P: 16, HC: 17 | 41.6 ± 9.5;  44.5 ± 9.6;  40.7 ± 10.4 | To investigate whether CFS patients without comorbid psychiatric diagnoses differ from CFS patients with comorbid psychiatric diagnoses and healthy control subjects in neuropsychological performance, the proportion with elevated spinal fluid protein or white cell counts, cerebral blood flow (CBF), brain ventricular lactate and cortical glutathione (GSH). | ^1^H MRS  ASL | in-house analysis toolkit |
| Okada T et al [63] | National Institute for Physiological Sciences | Japan | 2004 | CDC-Fukuda 1994 | 16;49 | 34.0 (24 -46); 34.4 (21 - 47) | To investigate brain structural changes in ME/CFS | sMRI | SPM2 VBM |
| Perrin et al [61] | University of Manchester | UK | 2010 | CDC-Fukuda 1994 | LD 1 year apart: 18; 9 | ME/CFS1*: 35.3 ± 12.6; ME/CFS2*: 36.1 ± 12.3; HC: 36.1 ± 12.4 | To investigate brain structural and structure change rate in ME/CFS | sMRI, FLAIR, MRA | Manually ventricle segmentation, Neuroradiologist assessment of deep white matter hyperintensity, semiautomatically determination of CBF and CSF flow |
| Peterson et al [70] | University of Minnesota Medical School | USA | 1994 | CDC-Holmes 1988 & NIH 1992 conference | 10;10 | 35.4 ± 9.5, 34.3 ± 8.3 | To test the hypothesis that mild exercise (walking 1 mph [1 mile = 1.609 km] for 30 min) would provoke serum cytokine and cerebral blood flow abnormalities of potential pathogenic importance in ME/CFS. | ^99^Tc^m^-HMPAO brain SPECT | Semiquantitative score by consensus of two radiologists |
| Puri et al [57] | Imperial College School of Medicine | UK | 2002 | CDC-Fukuda 1994 | 8;8 | 42.7 ± 8.4; 40.1 ± 8.8 | To test the hypothesis that ME/CFS is associated with altered cerebral metabolites in the frontal and occipital cortices. | MRS | proprietary Marconi (formerly Picker) software package |
| Puri et al [64] | Hammersmith Hospital | UK | 2012 | CDC-Fukuda 1994 | 26;26 | 42.9 ± 2.2; 38.2 ± 2.2 | To test brain structural differences in ME/CFS | sMRI | FSL-VBM |
| Schmailing et al [19] | University of Texas at El Paso | USA | 2003 | CDC-Fukuda 1994 | 15;15 | All: 44.4 ± 8.35 | To compare functional imaging among patients with ME/CFS and HC and to examine perceived and objective performance on PASAT. | ^99mTc^-ECD | Brain Registration and Analysis of SPECT Studies |
| Schwartz et al [18] | Brigham and Women's Hospital | USA | 1994 | CDC-Holmes 1988 & Oxford criteria | 16;15 (MRI), 14 (SPECT) | 42.5 ± 2.3; 39.2 ± 7.5 (MR); 49.6 ± 6.2 | To compare the usefulness of SPECT and sMRI in the detection of intracranial abnormalities in ME/CFS | PD MRI, T2 spin-echo MRI, and ^99^Tcm-HMPAO | Three radiologists blindly assess MR (hyperintensity on both PD and T2 SE) and SPECT (hypointensity) abnormalities |
| Sevel et al [65] | University of Florida | USA | 2018 | CDC-Fukuda 1994 | 18; 15 | 48.33 ± 12.67; 47.13 ± 11.67 | To evaluate using sMRI features for classification of ME/CFS | sMRI & RapidMiner | Indices of area, thickness, and volume for cortical structures and volume of subcortical structures rendered by FreeSurer, linear support vector machine from RapidMiner for classification |
| Shan et al [28] | Griffith University | Australia | 2018 | CDC-Fukuda 1994 | 45;27 | 47.12 ± 11.67; 43.10 ± 13.77 | To investigate functions of default mode network (DMN) in ME/CFS | Randomized event-related Stroop tfMRI and rsfMRI | FSL, SPM12, and in-house toolkit |
| Shan et al [29] | Griffith University | Australia | 2018 | CDC-Fukuda 1994 | 43;26 | 47.39 ± 11.81; 43.44 ± 13.93 | To investigate brain function characteristics during Stroop task | Randomized event-related Stroop tfMRI | FSL, SPM12, and in-house toolkit |
| Shan et al [47] | Griffith University | Australia | 2016 | CDC-Fukuda 1994 & Canadian 2003 | LD 6 years apart: 15;10 | 34.06 ± 8.77; 30.5 ± 7.93/6.43 ± 0.57; 6.21 ± 0.31 | To examine progressive brain changes associated with ME/CFS | sMRI, T1 SE, and T2 SE | SPM12 optimized VBM and voxel wise SE signal comparison |
| Shan et al [48] | Griffith University | Australia | 2017 | Canadian 2003 | 38;14 | 34.8 ± 10.1; 34.7 ± 8.4 | To examine brain structure variations associated with sleep quality in patients with ME/CFS | sMRI, T1 SE, MT-T1 SE, and T2 SE | SPM12 voxel wise signal comparison and regression |
| Shungu et al [51] | Weill Medical College of Cornell University | USA | 2012 | CDC-Fukuda 1994 | ME/CFS; MDD; HC: 15; 15; 13 | 32.7 ± 8.6; 31.7 ± 9.6; 27.6 ± 7.4 | To replicate cross-sectional elevations of ventricular lactate in ME/CFS and to explore possible reasons for lactate elevations | sMRI, proton MRSI, single slice ^31^P MRSI, single voxel proton MRS, pseudo-continuous ASL | Adapted in-house analysis procedure |
| Siessmeier et al [43] | Gutenberg University, | Germany | 2003 | CDC-Fukuda 1994 & NIH conference 1992 definition | 26;18 | 43 ± 9.3; 38 ± 11.5 | To evaluate cerebral glucose metabolism in ME/CFS patients | FDG-PET | three-dimensional stereotactic surface projection (3D-SSP, individual abnormality analysis software) and SPM99 for correlation analysis |
| Staud et al [73] | University of Florida | USA | 2018 | CDC-Fukuda 1994 & Canadian 2003 | 17;16 | 49.25 ± 11.43; 49.60 ± 10.00 | To evaluate CBF at rest, during task, and recovery abnormality in ME/CFS | pseudo-continuous arterial spin-labelling (pCASL) | SPM8 for preprocessing and ASLtbx for CBF map, prior selected ROI based analysis |
| Tanaka et al [40] | Osaka City University Graduate School of Medicine | Japan | 2006 | CDC-Fukuda 1994 | 6;7 | 30.4 ± 4.8; 26.1 ± 6.3 | To investigate brain responsiveness of ME/CFS patients during a fatigue-load | block design fMRI | SPM99 general linear model |
| Tirelli et al [37] | Centro di Riferimento Oncologico | Italy | 1998 | CDC-Fukuda 1994 | ME/CFS; MDD; HC: 18; 6; 6 | 34 ± 15, 48 ± 7, 38 ±1 2 | To investigate brain metabolism using FDG PET | ^18^FDG PET | ROI based quantitative analysis |
| van der Schaaf et al [15] | Radboud University Nijmegen | The Netherlands | 2017 | CDC-Fukuda 1994 & Reeves 2003 | 89;26 | 33.4 ± 1.2, 32.8 ± 2.1 | To test whether variations in DLPFC GMV and neuronal viability are associated with the defining clinical feature of ME/CFS (i.e., fatigue), or with cooccurring factors (e.g., pain, psychomotor speed, and physical activity) while taking into account the presence of depressive symptoms. | sMRI & single voxel MRS | SPM12 VBM & LCmodel for MRS |
| van der Schaaf et al [16] | Radboud University Nijmegen | The Netherlands | 2018 | CDC-Fukuda 1994 & Reeves 2003 | 85; 29 | 33.9 ± 1.2, 33.4 ± 2 | To quantify behavioural and neural correlates of state-related fatigue | event related fMRI | SPM12 two level GLM |
| Yamamoto et al [58] | Osaka City University Graduate School of Medicine | Japan | 2012 | CDC-Fukuda 1994 | Positive autoantibody against mAChR, ME/CFS (+), ME/CFS (-), HC:5, 6, 11 | 39.2 ± 7.0, 32.0 ± 2.5, 32.9 ± 6.5 | To investigate the effect of serum autoantibody of mAChR on brain functions | N-[^11^C] methyl-3-piperidyl benzilate ([^11^C] (+)3-MPB) and N-[^11^C] Methyl-4-piperidyl acetate ([^11^C]MP4A) PET | ROI based the Logan reference tissue method with the cerebellum as the reference region |
| Yamamoto et al [42] | Osaka City University Graduate School of Medicine | Japan | 2004 | CDC-Fukuda 1994 | 10;10 | 35.7 ± 8, 36.9 ± 10.1 | To assess the involvement of serotonin in the symptoms of chronic fatigue syndrome | [^11^C] (+) McN5652 PET | Voxel wise comparison of bind potential (BP) |
| Yoshiuchi et al [69] | UMDNJ-New Jersey Medical School | USA | 2006 | CDC-Fukuda 1994 | ME/CFS with psychiatric comorbidity, ME/CFS free from psychiatric comorbidity, HC 9, 16, 7 | 43.0 ± 7.1, 38.7 ± 6.5, 34.9 ± 9.9 | To test the hypothesis that patients with ME/CFS have reduced absolute CBF using Xenon computed tomography | Xenon-CT imaging | Modified Kety–Schmidt equation for CBF map and ROI based analysis |
| Zeinth et al [49] | Stanford University School of Medicine | USA | 2015 | Adapted CDC-Fukuda 1994 | 15;14 | 46.5 ± 13.2, 46.6 ± 14.6 | To (a) identify differences in gross brain structure in ME/CFS by using T1-weighted grey and white matter volumetric analysis, (b) detect microstructural abnormalities underlying ME/CFS by using DTI, and (c) detect global alterations in brain perfusion by using pseudo continuous arterial spin labelling (ASL). | sMRI, DTI, ASL |  |

**^§^**: N = Sample size (patients; controls).

*: NP = not provided; S1: ME/CFS patients with auditory information difficulties; S2: ME/CFS patients without auditory information difficulties; GAD: General Anxiety Disorder; MDD: major depression disorder; FM: fibromyalgia; LD: longitudinal design; ME/CFS1: ME/CFS patients with only osteopathic treatment; ME/CFS2: ME/CFS patients with treatment of their own choice;

**Table S2 Risk of bias of reviewed individual neuroimaging studies in myalgic encephalomyelitis/chronic fatigue syndrome (ME/CFS)^§^**

| Study | Patient Selection | Index Test | Control Standard | Confounding Factors |
| --- | --- | --- | --- | --- |
| Barnden et al [30] | 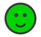 | 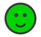 | 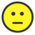 | 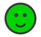 |
| Barnden et al [31] | 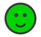 | 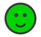 | 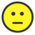 | 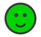 |
| Barnden et al [32] | 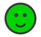 | 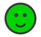 | 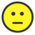 | 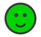 |
| Barnden et al [33] | 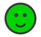 | 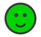 | 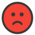 | 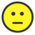 |
| Biswal et al [66] | 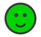 | 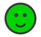 | 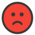 | 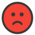 |
| Boissoneault et al [75] | 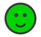 | 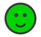 | 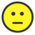 | 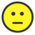 |
| Boissoneault et al [76] | 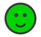 | 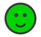 | 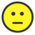 | 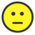 |
| Boissoneault et al [71] | 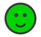 | 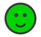 | 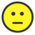 | 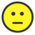 |
| Brooks et al [50] | 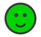 | 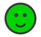 | 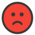 | 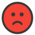 |
| Caseras et al [20] | 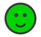 | 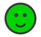 | 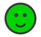 | 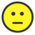 |
| Caseras et al [21] | 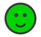 | 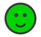 | 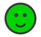 | 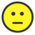 |
| Chaudhuri et al [53] | 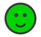 | 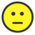 | 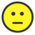 | 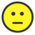 |
| Cleare et al [41] | 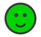 | 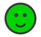 | 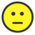 | 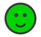 |
| Cook et al [44] | 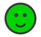 | 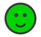 | 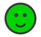 | 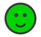 |
| Cook et al [22] | 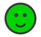 | 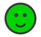 | 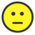 | 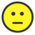 |
| Costa et al [17] | 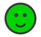 | 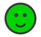 | 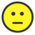 | 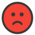 |
| De Lange et al [62] | 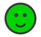 | 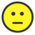 | 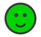 | 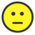 |
| De Lange et al [24] | 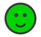 | 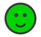 | 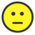 | 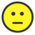 |
| Finkelmeyer et al [59] | 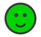 | 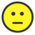 | 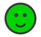 | 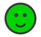 |
| Finkelmeyer et al [34] | 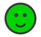 | 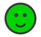 | 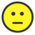 | 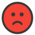 |
| Fischler et al [38] | 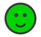 | 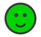 | 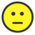 | 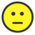 |
| Fischler et al [39] | 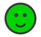 | 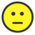 | 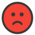 | 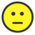 |
| Gay et al [74] | 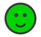 | 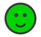 | 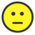 | 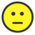 |
| Goldberg et al [67] | 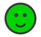 | 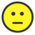 | 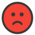 | 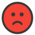 |
| He et al [60] | 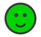 | 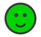 | NA | 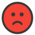 |
| Ichise et al [68] | 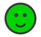 |  |  |  |
| Kim et al [13] |  |  |  |  |
| Kuratsune et al [36] |  |  |  |  |
| Lange et al [45] |  |  |  |  |
| Lange et al [25] |  |  |  |  |
| Lewis et al [72] |  |  |  |  |
| Mathew et al [54] |  |  |  |  |
| Miller et al [14] |  |  |  |  |
| Mizuno et al [26] |  |  |  |  |
| Mizuno et al [27] |  |  |  |  |
| Murrough et al [55] |  |  |  |  |
| Nakatomi et al [35] |  |  |  |  |
| Natelson et al [46] |  |  |  |  |
| Natelson et al [56] |  |  |  |  |
| Natelson et al [52] |  |  |  |  |
| Okada T et al [63] |  |  |  |  |
| Perrin et al [61] |  |  |  |  |
| Peterson et al [70] |  |  |  |  |
| Puri et al [57] |  |  |  |  |
| Puri et al [64] |  |  |  |  |
| Schmailing et al [19] |  |  |  |  |
| Schwartz et al [18] |  |  |  |  |
| Sevel et al [65] |  |  | NA | NA |
| Shan et al [28] |  |  |  |  |
| Shan et al [29] |  |  |  |  |
| Shan et al [47] |  |  |  |  |
| Shan et al [48] |  |  |  |  |
| Shungu et al [51] |  |  |  |  |
| Siessmeier et al [43] |  |  |  |  |
| Staud et al [73] |  |  |  |  |
| Tanaka et al [40] |  |  |  |  |
| Tirelli et al [37] |  |  |  |  |
| van der Schaaf et al [15] |  |  |  |  |
| van der Schaaf et al [16] |  |  |  |  |
| Yamamoto et al [58] |  |  |  |  |
| Yamamoto et al [42] |  |  |  |  |
| Yoshiuchi et al [69] |  |  |  |  |
| Zeinth et al [49] |  |  |  |  |

**Table S3 Observations of reviewed individual neuroimaging studies in myalgic encephalomyelitis/chronic fatigue syndrome (ME/CFS)^§^**

| Study | Observations |
| --- | --- |
| Barnden et al [30] | 1. T1 weighted spin echo (T1w SE) signal is elevated with increasing disease severity in the ventrolateral thalamus, internal capsule and prefrontal white matter (WM); 2. T2 weighted spin echo (T2w SE) signal decreased with increase of disease duration in right middle temporal WM; 3. T2w SE signal in right middle temporal WM in ME/CFS patients is greater that in healthy control (HC). |
| Barnden et al [31] | 1. WM volume in the midbrain decreases with increasing fatigue duration; 2. T1w SE signal and WM volume group × hemodynamic score interactions (abnormal ME/CFS correlation) were detected in the brainstem, deep prefrontal WM, the caudal basal pons and hypothalamus; 3. Grey matter (GM) volume in the brain stem is strongly correlated with pulse pressure in ME/CFS; 4. No global GM or WM volumetric difference was observed. |
| Barnden et al [32] | 1. Abnormal regressions of blood pressure (BP) and heart rate (HR) vs T1w SE signal were detected in nuclei of the brain stem vasomotor centre, midbrain reticular formation and hypothalamus, but also in limbic nuclei involved in stress responses and in prefrontal WM; 2. Regressions with BP and HR in the brain stem indicate involvement of the reticular formation, not WM tracts. |
| Barnden et al [33] | 1. Decreased T1w SE in brain stem, including hypothalamus, ventral tegmental area (VTA), medulla in ME/CFS; 2. Increased T1w SE in somatosensory areas in ME/CFS; 3. Inverse relationship between sensorimotor and brain stem T1w SE signals (myelin) was observed both in ME/CFS and HC. |
| Biswal et al [66] | 1. Whole cerebral blood flow (CBF) decrease in ME/CFS patients; 2. Regional CBF reduction in left (L)- and right (R)-frontal, LR-parietal, LR-temporal lobes; 3. Reductions of averaged CBF (ml/min/100 g tissue) in whole and regional brain regions; 4. 2 patients have increased CBF indicating ME/CFS is a heterogeneous illness |
| Boissoneault et al [75] | 1. ME/CFS patients have higher functional connectivity (FC) of bilateral superior frontal gyrus (SFG) with precuneus and postcentral gyrus, anterior cingulate cortex (ACC) with posterior cingulate cortex (PCC) and left thalamus/hippocampus, right angular gyrus (AG) with ipsilateral pre/postcentral gyri, and precuneus with bilateral supplementary motor area; 2. ME/CFS patients have lower FC in ACC, left parahippocampal gyrus, and bilateral pallidum to regions including right insula, right precentral gyrus, and hippocampus; 3. Connectivity of the left parahippocampal gyrus correlated strongly with overall clinical fatigue of ME/CFS patients. |
| Boissoneault et al [76] | 1. Static FC showed distinct changes during the paced auditory serial addition (PASAT) in participants: the insula and precuneus, increased in HC while decreased in ME/CFS; IFG, decreased in HC while slightly increased in ME/CFS. 2. Greater increases in inferior frontal gyrus (IFG) connectivity were associated with greater changes in fatigue rating as a result of PASAT performance; 3. HC had significantly higher dynamic FC than ME/CFS for connectivity between hippocampus and right superior parietal lobule. |
| Boissoneault et al [71] | 1. No whole CBF difference between ME/CFS and HCs nor correlation with fatigue level; 2. No heart rate variability (HRV) difference between ME/CFS and HC; 3. No significant difference of whole or regional variability in cerebral blood flow (CBFV) between ME/CFS and HC; 4. CBFV was inversely associated with fatigue rating; 5. Total power of HRV was negative correlated with fatigue; 6. Protective effects of high CBFV were greatest in individuals with low HRV. |
| Brooks et al [50] | 1. No significant difference in hippocampal volume between ME/CFS and HC; 2. Reduced but not significant difference of creatine/phosphocreatine (Cr) or choline-containing compounds (Cho) between ME/CFS and HC; 3. Significant reduction of N-acetylaspartate (NAA) in ME/CFS. |
| Caseras et al [20] | 1. No accuracy or response time difference in all n-back tasks between ME/CFS and HC; 2. Extra regions of bilateral medial and lateral prefrontal regions (BA10) and anterior cingulate gyrus (BA24/32) were recruited in ME/CFS patients during 1-back tasks; extra region of inferior/middle temporal gyrus (BA 21/37) was recruited in the 2-back and 3-back tasks in ME/CFS; 3. ME/CFS group showed reduced activation (percentage of BOLD signal change) in dorsolateral prefrontal (BA 10/45/46) and parietal (BA 7/19) cortices during the more demanding levels of the task; 4. During the 2-back and 3-back conditions, patients activated a large cluster in the right inferior/medial temporal cortex (BA 21/37), which was not activated by the control subjects. |
| Caseras et al [21] | 1. During the provocation of fatigue, ME/CFS patients showed significantly greater activation in in posterior brain regions, mainly occipital and parietal cortices, and posterior cingulate cortex than healthy controls; 2. In the anxiety-provoking condition, ME/CFS patients showed increased activations in both ventral (BA 10/11/47) and dorsal (BA 9/10/24/32) prefrontal regions compared with healthy controls; |
| Chaudhuri et al [53] | 1. Increased choline containing compounds (Cho) in the basal ganglia of ME/CFS. |
| Cleare et al [41] | 1. A widespread reduction in 5-HT1A receptor binding potential in ME/CFS; 2. A 23% reduction 5-HT1A receptor binding potential was observed in the hippocampus bilaterally. |
| Cook et al [44] | 1. ME/CFS and controls had similar HR, respiratory exchange ratio (RER) and lactate responses but lower oxygen consumption and work rate during exercise; 2. Cognitive performance was improved from pre- to post-exercise in controls but worsened in ME/CFS; 3. Brain responses to finger tapping did not differ between groups at either time point at preselected regions; 4. Patients exhibited increased brain activity from pre- to post-exercise compared to controls bilaterally for inferior and superior parietal and cingulate cortices for the Paced Serial Auditory Addition Task |
| Cook et al [22] | 1. Mental fatigue was significantly related to brain activity during the fatiguing cognitive task, positive correlations in cerebellar, temporal, cingulate and frontal regions and negative relationship in the left posterior parietal cortex; 2. ME/CFS participants did not differ from controls for either finger tapping or auditory monitoring tasks in prior selected regions, but exhibited significantly greater activity in several cortical and subcortical regions during the fatiguing cognitive task; 3. CFS participants were significantly slower and less accurate during the modified Paced Auditory Serial Attention test. |
| Costa et al [17] | 1. Lower perfusions (normalized to maximum activity count) in 24 ME/CFS patients (first group) were found in L and R frontal, R caudate, and brain stem; 2. Brain stem hypo perfusion was confirmed in all ME/CFS patients (67 patients). |
| De Lange et al [62] | 1. Both young and old cohorts of ME/CFS group showed significant reductions in the total GM volumes; 2. No regional GM difference was observed in either cohort; 3. No total or regional WM volume difference was observed; 4. The GM reduction was associated to the decline in physical activity. |
| De Lange et al [24] | 1. ME/CFS patients were considerably slower on performance of both motor imagery task and control visual imagery task; 2. ME/CFS patients solved the motor imagery task by recruiting additional cerebral regions supporting visual processes; 3. Ventral anterior cingulate cortex was active when healthy controls made an error but remained inactive when ME/CFS patients made an error. |
| Finkelmeyer et al [59] | 1. ME/CFS patients reported significantly higher levels of orthostatic intolerance (OI); 2. Higher severity of OI symptoms were associated with lower intra cranial compliance (ICC) and higher resting perfusion in ME/CFS; 3. In both ME/CFS and HC groups intracranial compliance was negatively correlated with cerebral perfusion; 4. There were no significant differences between the groups in intracranial compliance or perfusion. |
| Finkelmeyer et al [34] | 1. Accounting for total intracranial volume, patients had larger GM volume and lower WM volume; 2. Increased regional GM volume in several structures including the amygdala and insula in ME/CFS group; 3. Reductions in WM volume in the ME/CFS group were seen primarily in the midbrain, pons and right temporal lobe. |
| Fischler et al [38] | 1. Positive correlations were found between Hamilton Depression Rating Scale and rCBF in frontal lobes in ME/CFS; 2. There is neither a global nor a regional hypoperfusion in ME/CFS compared with HC. |
| Fischler et al [39] | 1. Three of 45 ROIs (L- and R- orbitofrontal and parietal inferior) showed lower trace uptake whereas 9 of ROIs (L- and R- frontal low, L- and R- prefrontal mid, L- and R- prefrontal high, L- prefrontal low, R- temporal lateral and frontal mid) showed higher trace uptake in ME/CFS than HC; 2. No subcortical hypoperfusion was noticed; 3. No significant correlations were found between frontal tracer uptake and CFS symptom behaviour measures; 4. Brain stem hyper perfusion in CFS without psychiatric morbidity as compared with HC and CFS with psychiatric morbidity. |
| Gay et al [74] | 1. No significant difference in global CBF between ME/CFS and HC; 2. Reduced regional resting CBF in occipital (right cuneus, inferior occipital gyrus, and left lingual gyrus) and temporal lobes (right parahippocampal gyrus) in ME/CFS; 3. ME/CFS patients showed disrupted intrinsic connectivity within the left frontoparietal network; decreased connectivity between the salience network and the left PCC; decreased connectivity in sensory motor network with midcingulate cortex; 4. No differences in FC were found within the DMN or RFPN; 5. The strength of connectivity between each of the disrupted regions was significantly correlated with self-reported fatigue ratings of the MFI; 6. The parahippocampal seed and three occipital lobe seeds showed altered FC with other brain regions. The degree of abnormal connectivity correlated with the level of self-reported fatigue. |
| Goldberg et al [67] | 1. Significant Hypoperfusion in L and R temporal lobes, L- and R- parietal lobes, and R- frontal lobe (Xe SPECT); 2. Significant hypoperfusion in bilateral orbitofrontal and anterior temporal, dorsal aspects of both frontal lobes and both parietooccipital lobes. |
| He et al [60] | 1. Significant inverse correlation was seen between CBF and skeletal muscle pH at rest; 2. Prolonged cerebral vascular constriction during the sympathetic phase of Valsalva manoeuvre (VM) was associated with higher pH in skeletal muscle after plantar flexion exercise. |
| Ichise et al [68] | 1. Forty-eight ME/CFS subject (80%) showed at least one or more decreased rCBF ratio (below 2 s.d. from the mean of NCs) below regions which include frontal (38 cases), temporal (21 cases), parietal (32 cases), and occipital ( 23 cases) lobes and basal ganglia (24 cases). |
| Kim et al [13] | 1. Increase connectivity from PCC (posterior cingulate cortex) to dorsal anterior cingulate (both right), rostral anterior cingulate cortex (right), middle temporal cortex (right), precuneus (left); 2. Lower global connectivity efficiency in PCC in CFS but no difference in local efficiency. |
| Kuratsune et al [36] | 1. H_2_^15^O PET identified lower global CBF and rCBF in multiple brain regions including frontal, occipital, temporal, subcortical, and brain stem; 2. A significant decrease of acetyl-carnitine was found in several regions of the brains of the patient group, namely, in the prefrontal (Brodmann’s area 9/46d) and temporal (BA21 and 41) cortices, anterior cingulate (BA24 and 33), and cerebellum. |
| Lange et al [45] | 1. No significant difference was found in the frequency of abnormal scans between ME/CFS and HC groups; 2. Disease duration was not significant related to presence of abnormalities; 3. Significant higher frequency of MR abnormalities (white matter hyperintensities-subcortical distinct) was found in CFS-No Psych (DSM III-R Axis-I) group compared with HC and CFS-Psych group. |
| Lange et al [25] | 1. Individuals with ME/CFS are able to process challenging auditory information as accurately as HCs but utilize more extensive regions of the network associated with the verbal WM system. 2. Individuals with ME/CFS appear to have to exert greater effort to process auditory information as effectively as demographically similar healthy adults. |
| Lewis et al [72] | 1. The twins with and those without ME/CFS were similar in mean number of visually detected abnormalities and in mean differences quantified by using image registration software. These results were unaltered with adjustments for fitness level, depression, and mood before imaging. |
| Mathew et al [54] | 1. Mean lateral ventricular lactate concentrations measured by 1H MRSI in ME/CFS were increased by 297% compared with those in generalized anxiety disorder (GAD) (P<0.001) and by 348% compared with those in healthy volunteers (P<0.001); 2. No ventricular volume difference; 3. Lactate concentration did not correlate with demographic variables, fatigue severity, or sleep quality; 4. Diagnosis of ME/CFS predicts lactate concentration. |
| Miller et al [14] | 1. ME/CFS patients exhibited significantly decreased activation (lower BOLD signal change amplitude) in the right caudate (p = 0.01) and right globus pallidus (p = 0.02); 2. Decreased activation in the right globus pallidus was significantly correlated with increased mental fatigue (r2 = 0.49, p = 0.001), general fatigue (r2 = 0.34, p = 0.01) and reduced activity (r2 = 0.29, p = 0.02) as measured by the Multidimensional Fatigue Inventory. |
| Mizuno et al [26] | 1. No difference in behaviour measures including response time; 2. BOLD signal change amplitude of the putamen was lower in the child ME/CFS group than in the HC group in the low-reward condition, but not in the high-reward condition; 3. BOLD signal changes of the putamen in the low-reward condition in child ME/CFS patients was negatively and positively correlated with severity of fatigue and the reward from learning in daily life, respectively. |
| Mizuno et al [27] | 1. Accuracy of picking out vowels (PV) plus story comprehension (SC) accuracy in ME/CFS were lower, other task performances including response time are similar; 2. Patients exhibited a much larger area of activation, recruiting additional frontal areas; 3. The right middle frontal gyrus (MFG), which is included in the dorsolateral prefrontal cortex, of child ME/CFS patients was specifically activated in both the single and dual tasks; this activation level was positively correlated with motivation scores for the tasks and accuracy of story comprehension; 4. In patients, the dorsal anterior cingulate gyrus (dACC) and left MFG were activated only in the dual task, and activation levels of the dACC and left MFG were positively associated with the motivation and fatigue scores, respectively. |
| Murrough et al [55] | 1. Increased ventricular lactate in ME/CFS compared with HC; 2. The mean ventricular volume did not differ between ME/CFS, major depressive disorder (MDD), or HC; 3. Within the ME/CFS group, there was a specific positive correlation between ventricular lactate and mental fatigue; 4. There was no main effect of group for water-normalized GABA or Glx in either the OCC or ACC voxels. |
| Nakatomi et al [35] | 1. There are no significant differences in cytokine (interferon-γ, interleukin-6, interleukin-1β, tumour necrosis factor-α) concentrations between groups; 2. ^11^C- (R)-PK11195 no displaceable binding potential values for ME/CFS patients were significantly higher in regions of midbrain, pons, thalamus, cingulate, hippocampus, and amygdala (in order of significance level between two groups); 3. ^11^C- (R)-PK11195 no displaceable binding potential values in the amygdala was positively correlated with cognitive impairment scores, those in hippocampus positively correlated with the Center for Epidemiological Studies depression scale. |
| Natelson et al [46] | 1. Higher frequency of abnormal scans (27%, hyperintensity foci on T2 scans from 9 patients and ventricular or sulcal enlargement in 5 patients) in ME/CFS patients compared with controls (2%); 2. Three patients with T2 hyperintensity developed other known neurological diseases. |
| Natelson et al [56] | 1. Mean CSF lactate levels in ME/CFS, fibromyalgia (FM) and ME/CFS+FM did not differ among the three groups but were all significantly higher than the mean values for control subjects. |
| Natelson et al [52] | 1. ME/CFS patient with and without psychiatric comorbidity did not show any difference in neuropsychological performance, the proportion with elevated spinal fluid protein or white cell counts, CBF, brain ventricular lactate and cortical glutathione (GSH). 2. The pooled ME/CFS patients showed lower GSH and CBF and higher ventricular lactate and rates of spinal fluid abnormalities than those in HCs. |
| Okada T et al [63] | 1. Patients with MW/CFS had reduced GM volume in the bilateral prefrontal cortex; 2. The volume reduction in the right prefrontal cortex paralleled the severity of the fatigue of the subjects. |
| Perrin et al [61] | 1. Mean proportionate CSF volume was not significantly different between ME/CFS and HCs; 2. All participants showed a slight increase in CSF between scans, but no significant difference was found between those with ME/CFS and those without; 3. No significant inter-group differences were found for any of the cerebral blood and CSF flow parameters; 4. Low levels of WMH were found in all participants. Objective scoring of WMH using Scheltens’ scale revealed no change in summary components; 5. No abnormal patterns in rate and extent of brain atrophy, ventricle volume, white matter lesions, cerebral blood flow or aqueduct CSF flow were detected in the ME/CFS population. |
| Peterson et al [70] | 1. In the ME/CFS group, 30% (3 of 10) of the patients had abnormal scans at rest, and 60% (6 of 10) had abnormal scans after exercise compared with HC 20% at rest and 20% after exercise although the difference after exercise is not statistically significant |
| Puri et al [57] | 1. The mean ratio of choline (Cho) to creatine (Cr) in the occipital cortex in ME/CFS was significantly higher than in the controls; 2. No NAA to Cr difference was observed. |
| Puri et al [64] | 1. Reduced GM volume in the ME/CFS group were noted in the occipital lobes (right and left occipital poles; left lateral occipital cortex, superior division; and left supracalcrine cortex), the right angular gyrus and the posterior division of the left parahippocampal gyrus; 2. Reduced WM volume in the ME/CFS group were noted in the left occipital lobe. |
| Schmailing et al [19] | 1. No group differences were found for performance on the PASAT despite ME/CFS perceived higher difficulty; 2. ME/CFS showed more diffused and less focal activation than HC; 3. Less rCBF in the anterior cingulate region during resting and task scans but significant greater increase in ME/CFS patients |
| Schwartz et al [18] | 1. 2.06 MRI abnormality foci per subject in ME/CFS vs. 0.80 foci per subject in HC although no significant difference; 2. SPECT abnormality 7.31 defects (hypoperfusion) per subject in ME/CFS vs 0.43 defects per subject in HC, significantly different. |
| Sevel et al [65] | 1. The sMRI model achieved 79.58% classification accuracy; 2. The self-report (accuracy=95.95%) outperformed the sMRI models; 3. Estimates from multiple brain areas related to cognition, emotion, and memory contributed strongly to group classification. |
| Shan et al [28] | 1. The ME/CFS patients required significantly longer time than NCs to perform the Stroop task; 2. ME/CFS patients have larger regions with increased blood flow and smaller regions with decreased blood flow when engaging in the Stroop task than NCs; 3. The SampEn (complexity) of the BOLD signal variation in the PCC was significantly higher in both tfMRI and rsfMRI in CFS patients; 4. In the resting state, FCs were significantly weaker in ME/CFS patients for mPFC–L-IPL and mPFC–R-IPL; 5. During the task, the FC for mPFC–PCC was significantly reduced relative to resting state in ME/CFS patients, whereas NCs maintained a similar connection strength. Thus, during the task, mPFC–PCC connectivity in ME/CFS was weaker than in NCs; 6. More complex dynamic FC among DMN hubs in ME/CFS. |
| Shan et al [29] | 1. No significant difference in BMI, HR, or PP between the ME/CFS and NC groups. The response time of ME/CFS patients was significantly longer than NCs; 2. ME/CFS patients have larger regions (93 areas extra) with greater blood flow change when engaging in the Stroop task than NCs; 3. The SampEns of BOLD signals in 10/50 areas activated in both ME/CFS and NCs were significantly lower in ME/CFS patients; 4. The SampEns of BOLD signals in 15 areas were significantly correlated with SF-36 physical component scores (PCS) and in 9 areas were significantly correlated with SF-36 mental component scores (MCS); 5. The SampEns of BOLD signals in the R vmPOS of the MVOcC accounted for 40% of variance in the SF-36 PCS and SampEns in the L A6cvl of the PrG accounted for an additional 16% in all subjects. The SampEns of BOLD signals in the R vmPOS of the MVOcC accounted for 31% variances in the SF-36 MCS and those in the L A6cvl of the PrG accounted for an additional 7% across all subjects. |
| Shan et al [47] | 1. A significant decrease in WM volumes in the left inferior fronto-occipital fasciculus (IFOF) in ME/CFS longitudinally while in NCs it was unchanged; 2. Pooled cross-sectional difference: decreased GM volumes in the right inferior temporal gyrus and increased in the right supplementary motor area; decreased WM volume in the left posterior part of IFOF/arcuate fasciculus; higher T1w signal intensities on the right in the parahippocampal gyrus, inferior temporal gyrus, and IFOF/arcuate fasciculus; higher T2w signal intensities in the right IFOF/arcuate fasciculus. |
| Shan et al [48] | 1. Regional WM volumes in the left inferior fronto‐occipital fasciculus (IFOF) were significantly lower, adjacent negative correlation between regional WM volumes and Pittsburgh sleep quality index (PSQI); 2. The magnetization transferring enhanced (MT) - T1w and T1w intensities were significantly and negatively correlated with PSQI in multiple regions, including the mPFC, internal capsule, and right IFOF. The secondary statistical assessment of inter‐group differences showed lower MT‐T1w and T1w intensities in the mPFC in ME/CFS; 3. T1w intensities in the right insula were significantly and negatively correlated with Chalder Fatigue Scale in ME/CFS. |
| Shungu et al [51] | 1. Elevated ventricular lactate in both ME/CFS and major depressive disorder (MDD) compared with HC; 2. Reduced occipital glutathione (GSH) level in ME/CFS and MDD; 3. Negative correlation between ventricular lactate and GSH across all participants, correlations were found between GSH and clinical variables and between lactate and clinical variables; 4. The ME/CFS group had lower rCBF values in the left anterior cingulate cortex and right lingual regions relative to the HC; 5. No significant phosphate level difference. |
| Siessmeier et al [43] | 1. 12 of 26 ME/CFS patients showed no significant difference; 12 ME/CFS patients showed decreased glucose metabolism in cingulate gyrus and adjacent mesial cortical areas, 5 of these 12 showed additional decrease in orbitofrontal/frontobasal cortex; 2 ME/CFS patients showed decreased glucose metabolism in cuneus/precuneus regions; 2. Group comparison showed decreased glucose metabolism in orbitofrontal cortex; 3. Correlation analyses showed significant correlations between some test scores (anxiety, depression, health related quality of life) but not fatigue and regional reductions in glucose metabolism. |
| Staud et al [73] | 1. Resting global CBF did not differ significantly between ME/CFS subjects and HC globally, in GM, or WM; 2. No clusters within the a priori ROI mask where CBF changes from the beginning to the end of the PASAT differed between ME/CFS subjects and HC; 3. During the recovery period, a small cluster within left inferior frontal gyrus was detected where rCBF increased in ME/CFS subjects but not controls; 4. The association between change in rCBF (bilateral STG and left cerebellum) from the beginning to the end of the PASAT and change in fatigue ratings differed significantly between ME/CFS and HC; 5. Correlations conducted by group indicated that reductions in rCBF in regions of left fusiform gyrus, R STG, L precuneus from the end of the PASAT to the end of the recovery period were associated with better recovery of fatigue for ME/CFS patients (negative correlation) whereas positive correlation was observed in HC. |
| Tanaka et al [40] | 1. Responsiveness of the task-dependent brain regions was decreased after the fatigue-inducing task in the normal and ME/CFS subjects and the decrement of the responsiveness was equivalent between the 2 groups; 2. In contrast, during the fatigue-inducing period, although responsiveness of auditory cortices remained constant in the normal subjects, it was attenuated in the ME/CFS patients. In addition, the rate of this attenuation was positively correlated with the subjective sensation of fatigue as measured using a fatigue visual analogue scale, immediately before the MRI session. |
| Tirelli et al [37] | 1. ME/CFS patients showed a significant hypometabolism in right mediofrontal cortex and brainstem in comparison with the healthy controls; 2. MDD patient showed a significant and severe hypometabolism of the medial and upper frontal regions bilaterally, whereas the metabolism of brain stem was normal. |
| van der Schaaf et al [15] | 1. Global GM volume and WM volume did not differ between the ME/CFS and healthy control groups; 2. Pain symptoms was the main predictor of both GM volume and NAA/Cr in the left dorsolateral prefrontal cortex of patients with ME/CFS. More pain was associated with reduced GM volumes and NAA/Cr; 3. No group difference in DLPFC NAA/Cr. |
| van der Schaaf et al [16] | 1. Patients with ME/CFS showed an effort-dependent directional behavioural bias toward less effort investment, which was accompanied by reduced feedback-related activity in the DLPFC for the highest effort level. |
| Yamamoto et al [58] | 1. Although the brain [11C] (+)3-MPB binding in ME/CFS (-), negative autoantibodies, patients did not differ from normal controls, ME/CFS (+), positive autoantibodies patients showed significantly lower [11C] (+)3-MPB binding than ME/CFS (-) patients and normal controls; 2. The [11C]MP4A index showed no significant differences among these three groups; 3. Neuropsychological measures were similar among groups. |
| Yamamoto et al [42] | 1. Significant reduction of the serotonin binding potential (BP) in the rostral subdivision of anterior cingulate (BA24/32) in chronic fatigue syndrome patients; 2. Pain score showed a negative correlation with the BP in anterior cingulate and other areas related to pain sensation, including cuneus precuneus, orbitofrontal cortex, posterior cingulate, and insular cortex. |
| Yoshiuchi et al [69] | 1. ME/CFS patients have diminished global CBF although ME/CFS patients with psychiatric comorbidity have reduced blood flow in right middle cerebral artery territory whereas those without have bilateral reductions. |
| Zeinth et al [49] | 1. There was significantly lower total supratentorial WM volume for patients with ME/CFS compared with control subjects; 2. Total and regional cortical GM volume was statistically equivalent; 3. Global and left hemispheric cortical thickness was equivalent between populations 4. Right lateral occipital, precentral, middle temporal, postcentral and pars orbitalis showed higher cortical thickness; 5. In the ME/CFS population, fractional anisotropy (FA) was increased in the right arcuate fasciculus, and in right-handers, FA was also increased in the right inferior longitudinal fasciculus; 6. In patients with CFS, right anterior arcuate FA increased with disease severity; 7. Arterial spin labelling (ASL) MRI showed no significant differences. |
